# Developing a hospital accreditation model: a Delphi study

**DOI:** 10.1186/s12913-021-06904-4

**Published:** 2021-08-27

**Authors:** Ali Mohammad Mosadeghrad, Fatemeh Ghazanfari

**Affiliations:** Department of Health Management and Economics, School of Public Health, Tehran University of Medical Sciences, Tehran, Iran

**Keywords:** Accreditation, Delphi study, Pluralistic evaluation, Model

## Abstract

**Background:**

Hospital accreditation (HA) is an external evaluation of a hospital’s structures, processes and results by an independent professional accreditation body using pre-established optimum standards. The Iranian hospital accreditation system faces several challenges. The overall aim of this study was to develop a model for Iran national hospital accreditation program.

**Methods:**

This research uses the modified Delphi technique to develop and verify a model of hospital accreditation. The first draft of the HA model was introduced through a critical review of 20 pioneer accreditation models and semi-structured interviews with 151 key informants from Public, private, semi-public, charity and military hospitals in Iran. Three rounds of Delphi were conducted with 28 experts of hospital accreditation to verify the proposed model. Panel members were selected from authors of research articles and key speakers in the area of hospital accreditation, senior managers of the country’s health system, university professors in the fields of health policy and management across the country.

**Results:**

A comprehensive model for hospital accreditation was introduced and verified in this study. The HA model has ten constructs of which seven are enablers (“Management and leadership”, “Planning”, “Education and Research”, “employee management”, “patient management”, “resource management”, and “process management”) and three are the results (“employee results”, “patient and society results” and “hospital results”). These constructs were further broken into 43 sub-constructs. The enablers and results scored 65 and 35% of the model’s total scores respectively. Then, about 150 accreditation standards were written and verified.

**Conclusions:**

A comprehensive hospital accreditation model was developed and verified. Proper attention to structures, processes and outcomes and systemic thinking during the development of the model is one of the advantages of the hospital accreditation model developed in this study. Hospital accreditation bodies can use this model to develop or revise their hospital accreditation models.

## Background

One of the main goals of a health system is to improve the quality and safety of hospital services. Large investments in the healthcare sector, rising demand, shortage of resources, increased medical errors, and the raising public expectations highlight the importance of controlling the quality and safety of hospital services provided to patients [1].

Hospital accreditation is the systematic evaluation and validation of a hospital by an independent external organization using a set of structural, process, and outcome standards [2]. An accreditation certificate ensures the quality of hospital care and is a key measure for hospital selection by patients, patient referral by physicians, and purchase of services from hospitals by health insurance organizations, especially in competitive environments [2].

Previous studies have reported different effects of hospital accreditation programs. Some studies have shown that these programs lead to the development of organizational policies and procedures [3], employee training [4], a healthy work environment [5], cooperation among employees [6], reduced conflict and better communications [7], increased responsibility [3], and higher job satisfaction [8]. Moreover, accreditation results in the development of hospital capacity [9] and its equipment [10], optimal use of hospital resources [10], higher quality of care [11, 12], safety [13, 14], effectiveness of hospital care [15, 16], reduction of medical errors [17–19], lower mortality rate [19], higher patient satisfaction [20], and, ultimately, better hospital performance [9–13]. In addition, receiving an accreditation certificate increases people’s confidence in the hospital and the quality of its services [4, 21], thus increasing its reputation and popularity [10].

On the other hand, some studies have questioned the usefulness of hospital accreditation programs. For example, a 2007 study on 37,000 patients in 73 hospitals across Germany showed that accreditation did not improve the quality of hospital care and did not increase patient satisfaction [22]. Another study on 36 hospitals in the US did not find a significant association between accreditation and reduction in medical errors [23]. Similarly, one study on Lebanese hospitals did not find a significant association between accreditation and patient satisfaction [17]. Some studies have shown that accreditation programs increase bureaucracy [7] and thus increase employee’s workload and resistance to the program [3] while increasing hospital costs [24]. The Office of Healthcare Accreditation of Iran’s Ministry of Health and Medical Education (MOHME) has planned a nationwide hospital accreditation program with the cooperation of Iran Universities of Medical Sciences. All the hospitals around the country are required to participate in this government-led program and receive certification [25]. Reimbursements to hospitals by health insurance organizations is based on their accreditation rating.

The first round of hospital accreditation took place in 2012–2013 using 8104 measurable elements for 38 hospital wards and the second round took place in 2013–2014 using 2157 measurable elements for 36 hospital wards [26]. The third round of hospital accreditation took place in 2016–2017 using 248 standards and 903 measurable elements under 8 constructs, and the fourth round has been ongoing in 2019–2020 using 110 standards and 514 measurable elements under 19 constructs [26, 27].

The first and second versions of the Iranian Hospital Accreditation Model were sectional. The third and fourth versions were functional models. Although this model has been revised four times between 2012 and 2020, there is still no logical link between its components, and not enough attention has been paid to its structures, processes, and outcomes. The fourth edition of the Iranian Hospital Accreditation Model includes the three Main-constructs, i.e., “management and leadership”, “care and treatment” and “service recipient”, and 19 secondary components with overlapping standards [28, 29].

Accreditation has had some benefits for Iranian hospitals. Improvement in hospital facilities and equipment, employee training, development of operational plans, formulation of policies and procedures for work processes, and customer orientation have been reported as some of the benefits of hospital accreditation in Iran [30, 31]. A 2015 study evaluated the hospital accreditation program in 547 Iranian hospitals and showed that 72% of the hospitals obtained a rating of 1 or higher in the first accreditation round [32]. However, there are studies that show that implementation of the accreditation program has not led to improvement in hospital performance [33–35], nor has it increased employee [36, 37] and patient [38] satisfaction.

The large number of standards, especially structural standards, vagueness of standards, overemphasis on documentation, impracticability of certain standards, inappropriate evaluation methods, low evaluation accuracy, surveyors’ lack of experience and skills and/or lack of independence, inconsistent evaluation procedures, and short-term certification are some of the challenges to the hospital accreditation program in Iran [26, 32, 39, 40].

**Fig. 1:**
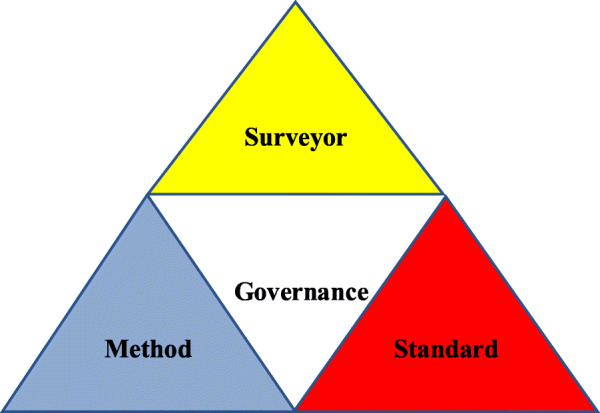
component of hospital accreditation

The hospital accreditation model and system play a key role in achievement of the objectives of accreditation programs. In general, hospital accreditation system consists of four components: governance, standard, method, and surveyor (Fig. 1).

Accreditation standards must be developed based on the principles of continuous improvement in order to enhance the quality of hospital care. Accreditation procedure must be designed in such a way as to ensure the quality, safety and effectiveness of hospital care while leading to the continuation of quality improvement programs in these hospitals. Surveyors must be qualified and evaluate hospital structures, processes, and outcomes based on a systematic and reliable method. Finally, the governance and management structure of the hospital accreditation program must be independent and reliable [25].

Deficiencies in governance structure, procedure, and/or surveyors lead to the failure of the hospital accreditation program to achieve its intended objectives. The results of a survey of 547 hospital managers across the country in 2015 showed that only about 10% were satisfied with the infrastructure of implementation of the hospital accreditation program in their hospitals. They complained about shortage of human, financial, and physical resources necessary to implement accreditation standards. Managers’ satisfaction with accreditation standards, procedure, and surveyors was average. 15.1 and 38% of the managers were satisfied with the content of the standards and the accreditation procedure respectively [41].

Another survey of hospital manager in Zanjan Province examined the effectiveness of Iran’s hospital accreditation program in 2016 and 2018. Hospital managers’ satisfaction with the accreditation system slightly decreased in the third accreditation round compared to the second round (by 0.66%). Their satisfaction with accreditation standard increased by 1.8%, but their satisfaction with accreditation procedure and implementation of standards decreased by 11.6 and 8.6% respectively. In other words, according to these hospital managers, the 2016 hospital accreditation program did not improve its performance compared to the previous rounds [42].

Accreditation standards must address inputs, outputs, and outcomes in a balanced manner to assist the accreditation program in achieving its ultimate goal. Models are a great tool for showing the relationships among the components of a phenomenon and shedding light on its complexities and blind spots. The purpose of the present research is to develop a model of the components of hospital accreditation standards as well as their relationships and the contribution of each standard to the achievement of accreditation objectives. The results can provide useful insights for hospital accreditation authorities in Iran and other countries and help in developing the appropriate model for hospital accreditation.

## Methods

This research uses the modified Delphi technique to develop and verify a model of hospital accreditation in Iran. The modified Delphi method was chosen because it allowed for expert interaction in the final round. This allowed members of the panel to provide further clarification on some matters and present arguments in order to justify their viewpoints. Studies have demonstrated that the modified Delphi method can be superior to the original Delphi method and perceived as highly cooperative and effective [43]. Also, In the classical Delphi technique, Expert Panel opinions are used to design an initial model in early stages, which is developed in later stages and presented to the expert panel to reach consensus. However, in the modified Delphi technique, an initial model is developed and then presented to the expert panel [44]. To develop the initial model of hospital accreditation, first a comparative review was conducted of the literature on accreditation models in 20 countries, including United States, Canada, Australia, Taiwan, Malaysia, New Zealand, South Korea, France, United Kingdom, Turkey, Denmark, Egypt, Lebanon, Saudi Arabia, Iran, India, Thailand, Indonesia, Zambia, and South Africa.

These are countries with a long history of hospital accreditation. Some of these models have been adapted by other countries into native accreditation models. An attempt has been made to select countries from each of the six WHO regions. Access to information was another criterion for country selection. A six-step protocol was used, including identification of countries, identification of areas under study, search for relevant documents, document selection, data extraction, and reporting of the findings.

First, information about the studied areas was collected by visiting the websites of accreditation agencies in the selected countries as well as the website of the International Society for Quality in Health Care (ISQua). Relevant articles were also extracted from valid databases and reviewed. A data collection form was used to collect data.

Areas of interest included the main and sub-constructs the models as well as the quantity and quality of standards and metrics. The search of English databases covered the period from 1990 to 2020. Gale’s seven stages of framework analysis were used to analyze the data. The results led to the identification of the codes that were used to develop the initial model [45].

Then, the strengths, weakness, and challenges of hospital accreditation standards in Iran were identified through interviews with 151 policymakers, managers and employees of MOHME’s Office of Supervision and Accreditation as well as Iran, Shahid Beheshti, Tehran, Tabriz, Isfahan, Yazd and Shiraz Universities of Medical Sciences, accreditation surveyors, managers and experts of health insurance organizations, and hospital managers and employee. The pluralistic evaluation approach was used and the interviewees were selected using purposive and snowball sampling techniques. Finally, grounded theory [46] was used to develop an initial model of hospital accreditation in Iran. Developing an initial model using a comprehensive literature review and presenting it to the expert panel reduces the stages of the Delphi technique and accelerates the process of achieving the final results.

The Delphi technique was used to verify the proposed initial model. The members of the Delphi panel must have in-depth knowledge of and differing perspectives on the issue under study and be highly credible in relevant scientific communities [47]. 28 individuals agreed to participate in the present research. The inclusion criteria for the expert panel invited to take part in the study were: Authors with at least three original research papers on hospital accreditation; keynote speakers in conferences on hospital accreditation; hospital CEOs and managers; and quality improvement managers as well as professors of health policy and management with at least 3 years of experience in accreditation. The expert panel with work experience in the field of accreditation were selected after reviewing their CVs. Authors of this article were excluded from this stage. The Delphi panellists’ key demographic characteristics are presented in (Table 1).

**Table 1:** Demographic characteristics of Delphi panel expert

Demographic variables	Frequency	Percentage
Gender		
Male	15	53/6
Female	13	46/4
Age		
30 to 40 years	14	50
41 to 50 years	13	46/5
51 years or older	1	3/5
Years of related experience		
3 to 5 years	8	28/6
6 to 10 years	13	46/4
11 to 15 years	6	21/5
16 to 20 years	1	3/5
Graduation degree		
Master of Science	2	7/2
Doctor of Philosophy	26	92/8
Occupation		
Accreditation Office Experts	4	14/3
Faculty members	14	50
Quality Improvement managers	10	35/7

92.8% of the participants had a PhD degree. The members of the expert panel had studied in various medical fields as well as health policy and management and health economics and were employed in the MOHME and Iran, Shahid Beheshti, Tehran, Tabriz, Isfahan, Yazd and shiraz universities of medical sciences

In the first stage of the modified Delphi approach, the initial hospital accreditation model was presented to the expert panel in the form of a questionnaire. This instrument had been reviewed by five professors in the field of health policy and management and its face and content validity had been established. The total average CVI was 0.96, which is acceptable.

This questionnaire provided the initial hospital accreditation model, including the main-constructs and sub-constructs of the model, the weight of the main-constructs, and the hospital accreditation standards. Each section contained items for obtaining the opinions of expert panel on the strengths and weaknesses of the proposed model, potential challenges to its implementations, and their recommended solutions. The opinions of the expert panel were analysed using thematic analysis. Quotations taken from the interview transcripts were labelled with the letter ‘E’. Finally, the proposed hospital accreditation model was modified based on the opinions of the expert panel.

In the second stage, the modified model of hospital accreditation in Iran was again presented to the expert panel in the form of a questionnaire to reach consensus. This approach is useful for converging expert panel opinions. First, a set of closed questions was used to ask expert panel about their agreement or disagreement with the key elements of the proposed model. These questions were rated on a 10-point Likert scale from 1 for ‘strongly disagree’ to 10 for ‘strongly agree’. Moreover, using an open question, experts who rated an item less than 7 were asked to explain their reasoning. The information obtained from the questionnaires was analysed in SPSS 24. Then, expert panel opinions were applied to the model. The adjusted model was presented to the expert panel in the third stage of the Delphi technique. The expert panel were asked to rate their agreement or disagreement with the key elements of the proposed model on a 10-point Likert scale from 1 for ‘strongly disagree’ to 10 for ‘strongly agree’. The role of the researchers was to study the comments of the expert panel and select the most repetitive suggestions for application in the initial and modified model in the second and third rounds of Delphi. The information was analysed in SPSS 24.

Measures of central tendency and dispersion, including mean, median, and standard deviation, were used to analyse the data obtained from the second and third rounds of the Delphi method. For all questionnaire items, the mean above 7 and the standard deviation less than 2, are the acceptable values for the model to be accepted by the expert panel.

This study formed a part of a PhD thesis in Tehran University of Medical Sciences. Ethical approval of the study was obtained from the University’s Research Ethics Committee (Ethics code: IR.TUMS.SPH.REC.1396.4870). The main ethical issues involved in this study were respondents’ rights to self-determination, anonymity and confidentiality. Respondents were given full information on the purpose and design of the study through a letter. Participants’ participation was voluntary and they could stop participating in the study at any point. Written and verbal informed consent was obtained from all participants. All methods were carried out in accordance with relevant guidelines and regulations.

## Results

**Fig. 2:**
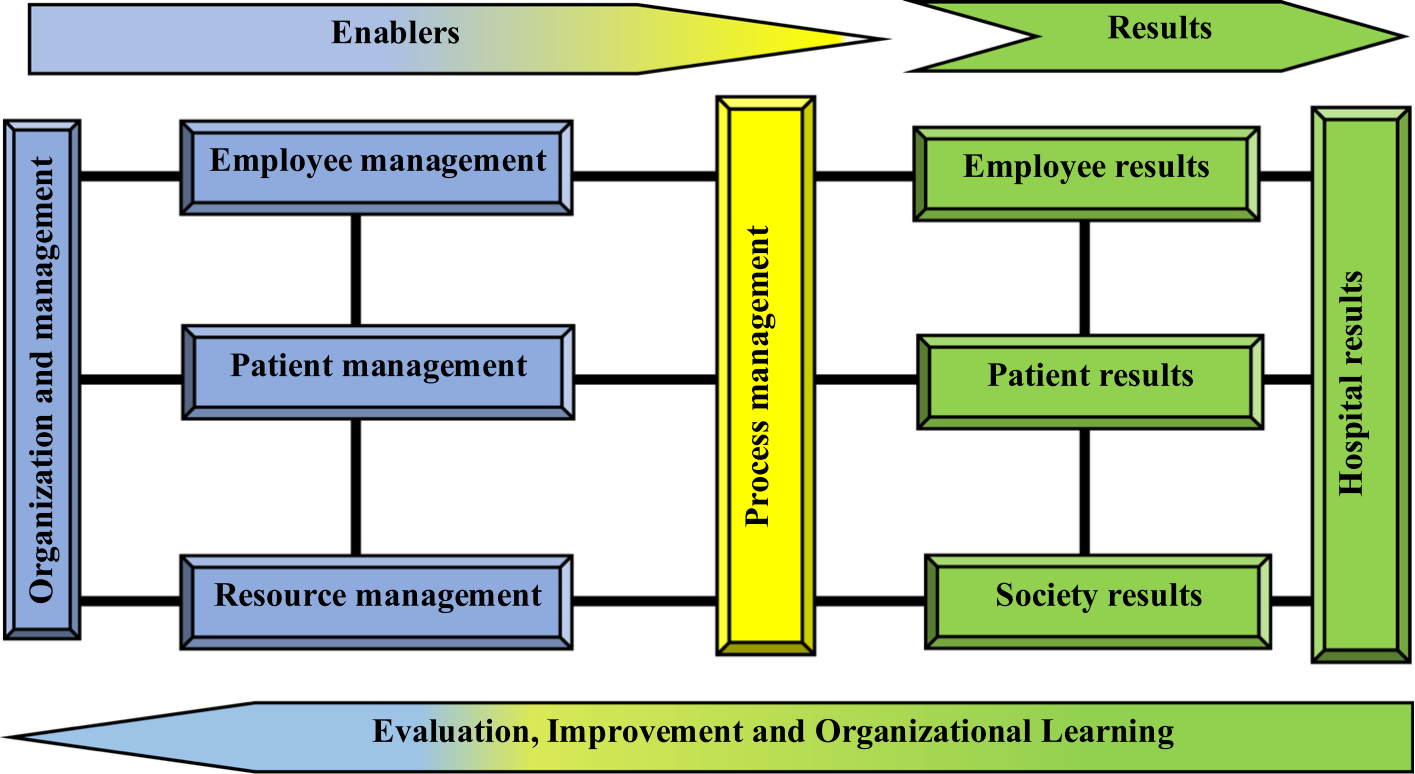
The initial proposed model of Iranian hospital accreditation

The initial hospital accreditation model was developed with 9 main constructs, including organization and management, employee management, patient management, resource management, process management, employee results, patient results, society results, and hospital results (Fig. 2), with 51 sub-constructs (Table 2) and 195 standards.

**Table 2:** Enablers and results of the initial proposed model of Iranian hospital accreditation

Enablers		Results
-Organizational governance - Executive management - Hospital committees - Crisis and disaster management - Strategic planning - Hospital Operational planning - Recruitment and job analysis - Performance appraisal and career development - Observance of employees’ rights - Management of buildings and facilities - nutrition management - hygiene and prevention - equipment, supplies and materials Management - Facility management - Financial management - Health information technology management - Patient reception- Patient evaluation- Patient care- Patient identification- medical services- Nursing services	- Continuity of care - Emergency services - Surgery and anesthesia services - Obstetrics and gynecology services - Dialysis services - Imaging services - Laboratory services - Blood transfusion medicine services - Psychiatric services - Physiotherapy services - Nutrition therapy services - Pharmaceutical services - Same service - Patient medical record - The patient falls - Transfer and discharge of the patient - Observing the rights of service recipients - Identify and document processes - Process evaluation - Process improvement	- Quality of working life - Job satisfaction of employees - Quality of health services - Patient satisfaction - Customer loyalty / re-election - hospital Clinical performance - hospital Operational performance - Hospital financial performance - hospital social responsibility

The proposed initial weights of the main-constructs are provided in Table 3. Initial weights indicate the impact of each of the main-constructs on the final outcome of hospital accreditation. The proposed weights are the result of brainstorming by the Iran’s accreditation experts in a meeting held at the headquarters of the Iran’s Ministry of Health and Medical Education. In this meeting, the experts of Iran Hospital Accreditation Program, including hospital accreditation officials, managers, university professors, surveyors, and standard setters, weighed the main-constructs.

**Table 3:** Weight of main constructs

Main constructs	Weight (percent)
Organization and management	12
Staff management	10
Patient management	12
Resource management	14
Process management	12
Staff results	10
Patient results	12
Society results	8
Hospital results	10

### Round 1

Every participant on the expert panel highlighted the necessity of developed main constructs. One university professor stated that, “in general, it’s a well-designed and comprehensive model” (E4). Another professor commented that “there are logical relationships among the components of the model” (E8). According to a faculty member, “the model is designed very well. It seems to be much more comprehensive and structured than the previous three hospital accreditation models” (E16). Similarly, the deputy director of treatment of one of the universities said: “the main and sub-constructs of this model are thorough and comprehensive” [E26]. These views were shared by another faculty member, who stated that “the relationship between the components of the model is logical and its special focus on outcomes is one of its strengths” (E1).

However, some of the participants made suggestions about how to improve the primary constructs of the proposed model. According to one university professor, “planning must be considered a separate main-construct. Planning is one of the main responsibilities of managers and separating its standards from the organization and management construct would highlight its importance” (E20). In addition, some interviewees argued that employee management must be combined with resource management, but they were persuaded after being presented with the reasons for such a distinction, including the importance and the distinctive nature of human resources and the high concentration of standards in the resource management construct. Another recommendation was to “combine society and patient results in the proposed model” (E6).

According to one of the hospital managers, the language of certain main-constructs needed to be modified: “I suggest using the phrase ‘management and leadership’ instead of ‘organization and management’ and ‘management of financial resources and costs’ instead of ‘financial management’” (E23). One faculty member suggested using “performance results instead of hospital results” (E4). Another faculty member said that “it is better to use the label customer or client management instead of patient management to include all clients” (E6). The explanation provided by the researcher was that the standards developed in this construct were related to patients and their companions. One quality manager stated that “it is better to use the phrase human capital management instead of employee management” (E9). The explanation provided against this argument was that the last words used in human resource management literature is employee management.

Moreover, the participants had ideas about changing the sub-constructs of the proposed model. One faculty member suggested that “the sub-constructs ‘quality improvement and patient safety’ and ‘infection prevention and control’ be added to the sub-construct ‘organization and management” (E20). According to some of the participants, education should have been considered as a separate main-construct. One hospital manager said that, “there should be education for all the groups that work in a hospital, both for employee and non- employee, and it is necessary to have education as a separate construct to illustrate its significance” (E15). The deputy director of treatment of one hospital argued that “the sub-constructs of strategic planning and operational planning are very broad and must be more descriptive” (E26).

One of the faculty members recommended that “the sub-construct of job analysis be removed from the sub-construct of employee management” (E27). Another faculty member recommended adding the sub-construct of “contribution management” that includes “issues of bidding, supervision, training, and suppliers” (E3). Of course, some experts believed that this is not recommended as currently universities are in charge of holding bids and hospitals practically play no important role in this process. The director of the accreditation office of a university stated that “environmental health and service recipient support can be added to the sub-constructs” (E11). The quality manager of a hospital recommended “adding a sub-construct for the main-construct ‘organization and management’ that includes decision making based on evidence and collective wisdom” (E2). Another quality manager recommended “separating health IT management from resource management and adding it to organization and management” (E8). According to one faculty member, “a sub-construct can be added the resource management section to include promotion of health and hygiene in the work environment” (E1). One university professor recommended removing “nutrition management and hygiene and prevention from the sub-constructs of resource management” (E10).

One of the participants mentioned that “it is better to combine the main-construct ‘facilities management’ with the sub-construct ‘buildings and facilities management’” (E24). Some of the participants believed that it was necessary to make changes in the composition of sub-constructs within the main-construct of patient management. According to one faculty member, “sub-constructs of patient management must be worded more broadly. The sub-construct ‘imaging and laboratory’ can be labelled ‘paraclinical services’. The sub-construct ‘patient falls’ can be removed. sub-constructs ‘medical care’ and ‘nursing care’ can be removed and the standards within these content can be listed under the sub-construct ‘general patient care’. Similarly, standards within the sub-construct ‘psychiatric services’ and ‘physiotherapy’ can be listed under the broader sub-construct of ‘general patient care’” (E17).

In addition, the experts on the panel were also inquired about the weight of the main- constructs. They evaluated the weight ratio of enablers to results to be appropriate. One of the faculty members stated that “there is good balance between the inputs and outputs of the model” (E7). However, some of the participants discussed their suggestions about the weight of main-constructs. One faculty member argued that “it is better to reduce the weight of ‘resource management’ and add to the weight of ‘employee management’” (E13). According one of the hospital managers, “the weight of the ‘patient management’ construct is too high given the weights assigned to patient and society results” (E23).

**Fig. 3:**
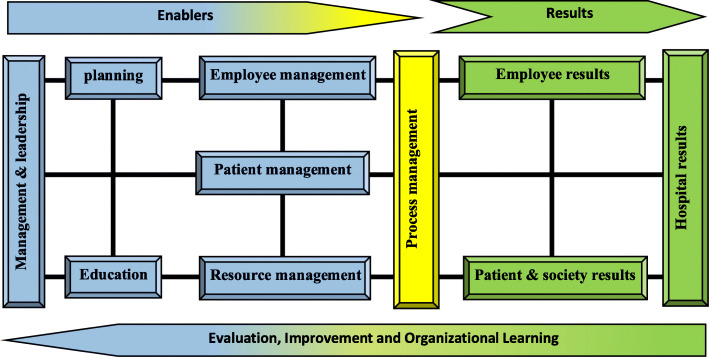
The modified proposed model of Iranian hospital accreditation

The expert’s opinion was considered and the modified model including 10 constructs namely *management and leadership*, *planning*, *education*, *employee management*, *resource management*, *patient management*, *process management*, *employee results*, *patients and society results* and *hospital results* (Fig. 3) and 37 sub-constructs (Table 4) were developed. The second round Delphi was held with 28 experts.

**Table 4:** Main-constructs, sub-constructs and weights of proposed model of accreditation

Main constructs	Weight	Sub-constructs	Main constructs	Weight	Sub-constructs
Management & leadership	12%	- Organizational governance - Executive Management - Patient quality and safety improvement - Prevention and control of infection - Hospital committees - Crisis and disaster management	**Planning**	10%	- Vision, mission and values of the organization - Goals and objectives - Strategies - Action plan - Performance indicators
Education	8%	- Employee education - Patient education - Contractors education	**Employee management**	8%	- Job analysis - Staff recruitment - Performance appraisal and career development - Observance of employees’ rights
Resource management	9%	- Buildings & facilities management - Equipment, supplies & materials Management - Health information technology Management - Financial Management	**Patient management**	8%	- Patient reception - Patient evaluation - Patient care General patient care Emergency services Surgery and anesthesia services Intensive care Continuity of care paraclinical services Blood transfusion Medicine services Rehabilitation services Nutrition services Pharmaceutical services Organ donation Patient medical record Observance of the rights of service recipients - Patient discharge
Process management	10%	- Identification and documentation of processes - Process evaluation - Process improvement			
Employee results	11%	- Quality of personal and professional life - Organizational commitment of employees			
Patient and society results	12%	- Quality of care and patient safety - Patient / recipient satisfaction - Social responsibility of the hospital			
Hospital results	12%	- hospital Clinical performance - hospital Operational performance - Hospital financial performance			

### Round 2

In the second Delphi stage, the experts were asked to rate their agreement with the items on a 10-point Likert scale from 1 for ‘totally disagree’ to 10 for ‘totally agree’. They were also asked to explain their reasoning for rating any item below 7. The results of statistical analysis indicated that the experts approved of all the main-constructs of the proposed model. In addition, the participants emphasized that the construct of education should be changed to ‘education and research’. One of the hospital managers said: “a research construct must be added to the main constructs of the model or you can consider education and research as a separate main construct that contains all the standards related to research and education” (E14). The results also highlighted the need for making changes in the composition of the sub-constructs. The results of statistical analysis in the second stage of the Delphi technique are provided in Table 5.

**Table 5:** The Statistical Result for HA Model, main-construct, sub-construct and weight of HA Model in Second and third Round of Delphi Study

Constructs	Question	Median	Mean	SD	consensus	Median	Mean	SD	consensus
		Results of round two				Results of round three			
Management and leadership	Do you consider the existence of “management and leadership” appropriate?	9	8.01	0.73	✓	9	8.11	0.69	✓
	Is the proposed weight of “management and leadership” appropriate?	9	8.42	1.01	✓	9	8.57	1.12	✓
	Is there a logical connection between the “management and leadership” construct and its sub- constructs?	8	6.79	1.87	×	9	8.20	0.73	✓
	Do “management and leadership” sub- constructs cover all aspects of this constructs?	8	6.42	1.23	×	9	8.15	0.79	✓
	Is there a logical connection between “management and leadership” sub- constructs and its standards?	7	5.23	1.78	×	9	8.43	0.84	✓
	Do “management and leadership” standards cover all aspects of this constructs?	7	5.76	1.98	×	9	7.76	0.93	✓
Planning	Do you consider the existence of “planning” appropriate?	9	8.12	1.02	✓	9	8.08	0.98	✓
	Is the proposed weight of “planning” appropriate?	9	7.53	0.76	✓	9	7.68	0.89	✓
	Is there a logical connection between the “planning” construct and its sub- constructs?	8	6.65	1.93	×	9	8.35	0.87	✓
	Do “planning” sub- constructs cover all aspects of this constructs?	8	5.44	1.53	×	9	8.19	0.64	✓
	Is there a logical connection between “planning” sub- constructs and its standards?	8	5.71	1.69	×	9	8.71	0.73	✓
	Do “planning” standards cover all aspects of this constructs?	8	7.07	1.72	✓	9	8.54	0.87	✓
Education	Do you consider the existence of “Education” appropriate?	8	7.73	1.13	✓	–	–	–	–
	Is the proposed weight of “Education” appropriate?	9	7.5	0.9	✓	–	–	–	–
	Is there a logical connection between the “Education” construct and its sub- constructs?	8	6.78	1.64	×	–	–	–	–
	Do “Education” sub- constructs cover all aspects of this constructs?	7	4.71	2.61	×	–	–	–	–
	Is there a logical connection between “Education” sub- constructs and its standards?	8	7.59	0.76	✓	–	–	–	–
	Do “Education” standards cover all aspects of this constructs?	7	6.64	0.64	×	–	–	–	–
Education & research	Do you consider the existence of “Education & research” appropriate?	–	–	–	–	8	7.98	1.02	✓
	Is the proposed weight of “Education & research” appropriate?	–	–	–	–	9	7.74	0.73	✓
	Is there a logical connection between the “Education & research” construct and its sub- constructs?	–	–	–	–	9	8.95	0.79	✓
	Do “Education & research” sub- constructs cover all aspects of this constructs?	–	–	–	–	9	8.67	0.65	✓
	Is there a logical connection between “Education & research” sub- constructs and its standards?	–	–	–	–	9	8.53	0.81	✓
	Do “Education & research” standards cover all aspects of this constructs?	–	–	–	–	9	8.46	0.84	✓
Employee management	Do you consider the existence of “Employee management” appropriate?	9	7.14	1.09	✓	9	7.65	0.87	✓
	Is the proposed weight of “Employee management” appropriate?	9	7.46	0.94	✓	9	7.54	0.83	✓
	Is there a logical connection between the “Employee management” construct and its sub- constructs?	8	7.54	0.73	✓	9	8.5	0.85	✓
	Do “Employee management” sub- constructs cover all aspects of this constructs?	9	7.42	1.01	✓	9	8.42	1.01	✓
	Is there a logical connection between “Employee management” sub- constructs and its standards?	9	7.98	0.85	✓	9	8.12	0.67	✓
	Do “Employee management” standards cover all aspects of this constructs?	9	7.69	0.91	✓	9	8.39	0.72	✓
Patient management	Do you consider the existence of “Patient management” appropriate?	10	10	0	✓	10	10	0	✓
	Is the proposed weight of “Patient management” appropriate?	8	7.39	1.11	✓	8	7.62	0.91	✓
	Is there a logical connection between the “Patient management” construct and its sub- constructs?	9	6.67	1.89	×	9	8.60	1.02	✓
	Do “Patient management” sub- constructs cover all aspects of this constructs?	9	6.42	2.17	×	9	8.57	1.12	✓
	Is there a logical connection between “Patient management” sub- constructs and its standards?	10	7.60	1.14	✓	9	8.73	1.17	✓
	Do “Patient management” standards cover all aspects of this constructs?	10	7.39	1.07	✓	9	8.15	1.15	✓
Resource management	Do you consider the existence of “Resource management” appropriate?	10	7.81	1.23	✓	10	7.74	1.12	✓
	Is the proposed weight of “Resource management” appropriate?	9	7.53	0.77	✓	9	7.53	0.77	✓
	Is there a logical connection between the “Resource management” construct and its sub- constructs?	10	7.92	1.01	✓	9	7.91	1.1	✓
	Do “Resource management” sub- constructs cover all aspects of this constructs?	9	6.93	1.12	×	9	7.89	1.12	✓
	Is there a logical connection between “Resource management” sub- constructs and its standards?	9	7.57	0.82	✓	9	8.54	0.75	✓
	Do “Resource management” standards cover all aspects of this constructs?	9	7.42	0.86	✓	9	8.33	0.89	✓
Process management	Do you consider the existence of “Process management” appropriate?	10	8.78	1.45	✓	10	8.78	1.45	✓
	Is the proposed weight of “Process management” appropriate?	9	8.64	0.61	✓	9	8.58	0.75	✓
	Is there a logical connection between the “Process management” construct and its sub- constructs?	6	4.25	2.15	×	9	8.34	1.12	✓
	Do “Process management” sub- constructs cover all aspects of this constructs?	6	4/08	2/17	×	9	8/14	1/06	✓
	Is there a logical connection between “Process management” sub- constructs and its standards?	6	6/63	1/54	×	9	8/34	1/15	✓
	Do “Process management” standards cover all aspects of this constructs?	6	4.58	1.71	×	9	7.97	0.97	✓
Employee results	Do you consider the existence of “Employee results” appropriate?	9	7.73	1.44	✓	9	7.73	1.44	✓
	Is the proposed weight of “Employee results” appropriate?	9	7.28	1.09	✓	9	7.43	0.89	✓
	Is there a logical connection between the “Employee results” construct and its sub- constructs?	9	7.21	0.97	✓	9	8.39	0.45	✓
	Do “Employee results” sub- constructs cover all aspects of this constructs?	9	8.03	1.08	✓	9	8.03	0.79	✓
	Is there a logical connection between “Employee results” sub- constructs and its standards?	9	8.14	0.98	✓	9	8.15	0.73	✓
	Do “Employee results” standards cover all aspects of this constructs?	9	8	1.10	✓	9	7.99	0.59	✓
Patient & society results	Do you consider the existence of “Patient & society results” appropriate?	9	7.94	1.14	✓	9	7.81	1.25	✓
	Is the proposed weight of “Patient & society results” appropriate?	9	7.32	1.10	✓	9	7.42	1.18	✓
	Is there a logical connection between the “Patient & society results” construct and its sub- constructs?	9	7.46	0.86	✓	9	8.44	0.86	✓
	Do “Patient & society results” sub- constructs cover all aspects of this constructs?	9	7.79	0.93	✓	9	8.35	0.93	✓
	Is there a logical connection between “Patient & society results” sub- constructs and its standards?	9	7.86	0.89	✓	9	8.39	0.77	✓
	Do “Patient & society results” standards cover all aspects of this constructs?	9	7.98	0.92	✓	9	8.24	0.42	✓
Hospital results	Do you consider the existence of “Hospital results” appropriate?	9	7.89	0.87	✓	9	7.46	1.21	✓
	Is the proposed weight of “Hospital results” appropriate?	9	7.32	1.10	✓	9	7.22	1.15	✓
	Is there a logical connection between the “Hospital results” construct and its sub- constructs?	9	8.14	0.95	✓	9	8.18	0.73	✓
	Do “Hospital results” sub- constructs cover all aspects of this constructs?	9	8	1.13	✓	9	8	1.13	✓
	Is there a logical connection between “Hospital results” sub- constructs and its standards?	9	8.21	0.93	✓	9	8.29	0.79	✓
	Do “Hospital results” standards cover all aspects of this constructs?	9	8.16	0.97	✓	9	8.27	0.86	✓

### Round 3

Experts’ suggestions about required changes were applied to the model and the modified model was developed with 10 constructs and 43 sub-constructs. The third round of Delphi was done like the second round of Delphi. Statistical results including average and standard deviation for the proposed hospital accreditation model in third Round of Delphi Study are shown in Table 5.

### Final model

**Fig. 4:**
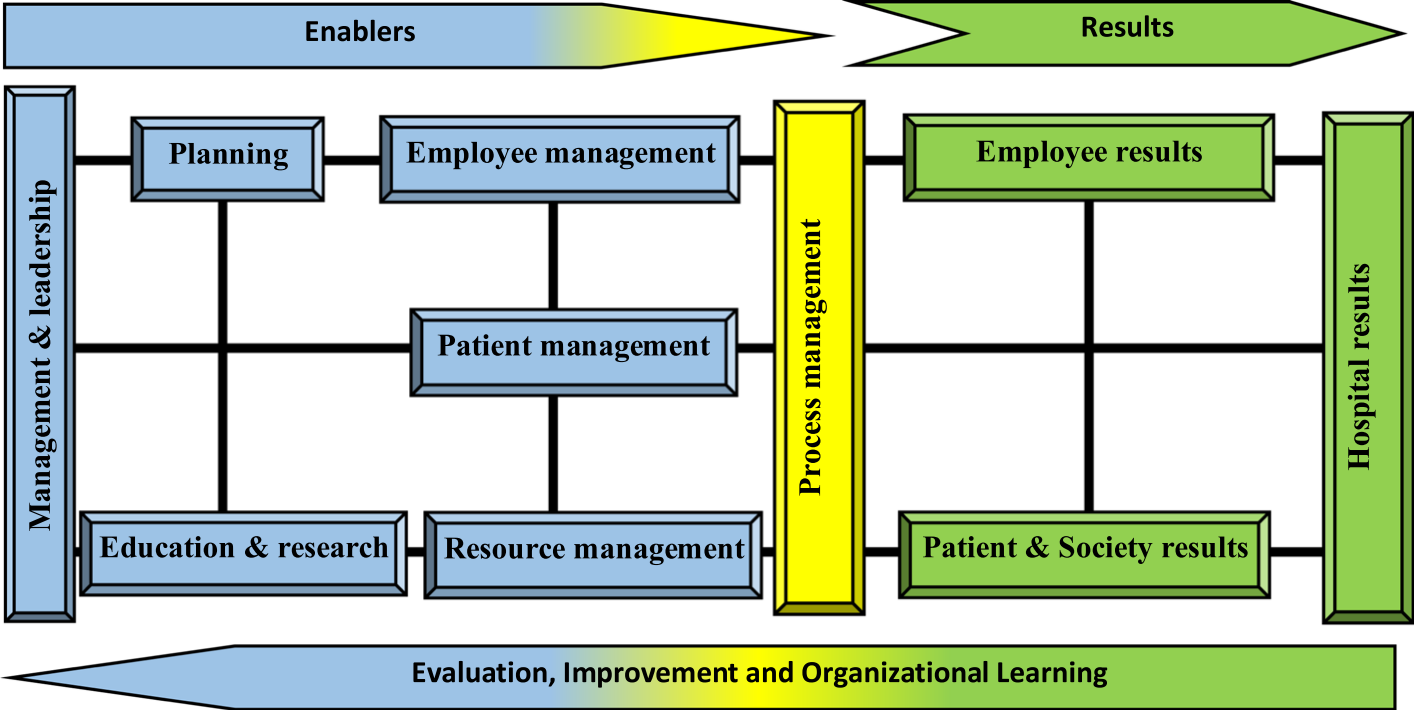
Hospital Accreditation (HA) model

The third round of Delphi showed that the proposed model is comprehensive and applicable to hospitals. Figure 4 showed the final Iranian hospital accreditation model. Results of main-constructs’ weights and final sub-constructs are shown in Table 6.

**Table 6:** Final main-construct, weight and sub-construct of HA model

Main constructs	Weight	Sub-constructs	Main constructs	Weight	Sub-constructs
Management & leadership	12%	Organizational governance Executive Management leadership disaster risk management	**planning**	10%	Hospital Goals Hospital operational planning
Education and Research	8%	Hospital educational planning Practical hospital research	**Employee management**	8%	employee recruitment employee Compensation employee Performance evaluation
Resource management	9%	Buildings & facilities management Equipment & supplies management Health information Technology management Financial management	**Patient management**	8%	Patient reception Inpatient services Outpatient services Emergency services Surgery and anesthesia services Intensive care Obstetrics and Gynecology Services Dialysis services Paraclinic services Blood transfusion medicine services Rehabilitation services Nutrition services Pharmaceutical services Continuity of care Organ donation Observance of the rights of patient Patient discharge
Process management	10%	Processes Identification Patient quality and safety improvement Prevention and control of infection			
Employee results	11%	Employee job satisfaction Employee Organizational commitment			
Patient & society results	12%	Quality and safety of hospital services Satisfaction of patients and companions Social responsibility of the hospital			
Hospital results	12%	Hospital Clinical performance Hospital Operational performance Hospital financial performance			

The experts were also surveyed about the developed standards. Their opinions were applied to the model after each Delphi stage and the adjusted standards were presented to the panel of experts for feedback. After three Delphi stages, the panellists approved a total of 153 standards for hospital accreditation in Iran. The number of standards of each main construct are shown in Table 7.

**Table 7:** Number of standards of Iranian hospital accreditation model

Enablers							
Main constructs	Management & leadership	Planning	Education & research	Employee management	Patient management	Resource management	Process management
Number of standards	16	6	12	10	60	20	13
Results							
Main constructs	Employee results		Patient & society results			Hospital results	
Number of standards	5		5			6	

## Discussion

A comprehensive model for hospital accreditation standards was introduced and verified in this study. The HA model has ten constructs of which seven are enablers (“Management and leadership”, “Planning”, “Education and Research”, “employee management”, “patient management”, “resource management”, and “process management”) and three are the results (“Employee results”, “patient and society results” and “Hospital results”). These constructs were further broken into 43 sub-constructs (Table 6).

The proposed hospital accreditation model is a comprehensive and integrated model. The dominant logic of the model is the development of enablers that will lead to desirable outcomes through certain processes and with optimal utilization of resources. According to the Donabedian model, information about quality of care can be drawn from three categories: “structure,” “process,” and “outcomes.” Structure describes the context in which care is delivered, including hospital buildings, staff, financing, and equipment. Process denotes the transactions between patients and providers throughout the delivery of healthcare. Finally, outcomes refer to the effects of healthcare on the health status of patients and populations [48].

“Management and leadership” is the top enabler in this hospital accreditation model in terms of importance. The success or failure of any organization depends to a large extent on its system of management and leadership. Due to the specialized nature of hospital care, hospital managers must be intelligent, knowledgeable, judicious, competent, experienced, and committed [1]. Leadership is the science and art of influencing, persuading, and mobilizing employees to achieve organizational goals [1]. Commitment and involvement of managers and their ability to motivate employees to accomplish intended goals are crucial to the success of the hospital accreditation model. In their leadership role, managers are responsible to empower and guide employees to achieve the mission of the organization and bring about the changes that are necessary to accomplish its vision. Therefore, hospital managers can motivate employees to achieve the vision and goals of the organization by developing a challenging vision and adopting transformational and participatory leadership and facilitate this process by providing the required resources and effective guidance to the employees [49]. Acceptance of the accreditation program by the employees depends on managers’ attitude toward accreditation. Committed managers can build employees’ commitment to implementing the accreditation program [50, 51].

Planning plays a significant role in accomplishing the established goals. Quality should be recognized as an organization’s strategic goal and should be reflected in the organization’s corporate vision and mission [52]. Planning ensures that the resources needed to achieve these goals are correctly identified and made available at the right time. It also prevents duplication of effort and wasting of resources. In the proposed hospital accreditation model, standards in the planning construct are organized under two sub-constructs, i.e., hospital goals and operational plan. These standards focus on the analysis of internal and external factors to identify the strengths and weaknesses of the hospital, set goals, develop operational plans, establish the desirable level of key performance indicators, and measure the effectiveness of operational plans.

Training and education are key components in a quality management program, and have an important role in establishing a common language of quality, and securing commitment and behaviour change toward continuous quality improvement. Education and training enhance employees’ job-related skills, communication and teamwork and help overcome employees’ resistance to quality management change [53]. Healthcare managers should develop the technical capabilities of employees and enable them to improve the quality of services continuously. Education and training provide the necessary knowledge, skills and abilities for employees to do the right job effectively. Increased training in job skills results in improved processes which improve product quality [54]. Education goals must be aligned with organizational goals.

Also, large amounts of data are generated in hospitals, which must be used correctly as per the accreditation standards. Health research has high value to society. It can provide important information about disease trends and risk factors, outcomes of treatment or public health interventions, functional abilities, patterns of care, and health care costs and use [55, 56]. Collectively, health research has led to significant discoveries, the development of new therapies, and a remarkable improvement in health care and public health. Due to its significance, a separate main-construct is dedicated to education and research.

Employee management is one of the key responsibilities of hospital managers, since hospitals are complex systems with conservative staff that tend to resist organizational changes. Hospital staff must be highly flexible, continuously interact with one another, and adapt to all organizational changes. Implementation of accreditation programs requires significant changes in hospitals, which is highly likely to be resisted by the staff. People are the drivers of any organizational change. The effectiveness of an organization depends on the extent to which people perform their roles and move towards the corporate goals and objectives [57]. Therefore, effective employee management, from selection and deployment to compensation and job satisfaction, is crucial to the achievement of accreditation objectives.

Provision of high-quality and safe care to patients is the foundation of hospital accreditation. In other words, quality improvement programs such as accreditation cannot succeed without focusing on patients. Systems and processes must be in place to identify customer needs, translate these needs into appropriate organizational requirements and satisfy them [58]. The ultimate measure of an organization’s performance is customer satisfaction [59]. While satisfied customers may tell only four or five people about their experience, dissatisfied customers will tell 20 people [60]. Therefore, it is very important to find customer satisfaction and customer perception of quality. The insights gained can clearly help the organization improve quality. Patient management encompasses all the services provided to patients from admission to the hospital to delivery of care, discharge, and even through convalescence. The goal of hospitals is to provide high-quality and safe diagnostic, therapeutic, rehabilitative, and palliative care to patients.

Allocation of resources (i.e., human, equipment, and material) is necessary for delivering desired healthcare services to meet customer needs. Managers are responsible to provide appropriate resources to make the implementation of quality management successful. The effectiveness of quality management arises from top management efforts towards the creation of supportive organizational structures and systems to manage the organization’s quality journey and facilitate the implementation of quality management strategy across departments [51, 61, 62]. Hospital management must ensure the availability and safety of required resources in order to achieve the objectives of the accreditation program. In addition, the environment for provision of care must be safe for both the providers and recipients of healthcare services. This construct also addresses the quality of suppliers and contractors.

Emphasis should be placed on improving the processes rather than on blaming employees [63]. Therefore, quality must be designed into the processes. A process is a collection of activities that transforms inputs into an output (product or service). Effective management, continuous improvement, and regular evaluation of hospital processes contributes to the success of hospital accreditation programs. The high number of wards in hospitals and their interrelationships may hinder hospital accreditation. Various studies have reported the high number and complexity of work processes in hospitals, poor coordination between wards and units, and lack of clear mechanisms for monitoring and evaluation of processes as barriers to the achievement of hospital accreditation objectives [64]. Hospital processes must be enhanced so that quality, safe, and effective care is provided to patients. Achievement of all the objectives of hospital accreditation is contingent on processes through which activities are performed and goals are accomplished. Therefore, it is necessary to identify key processes and control and improve their quality and safety. Precisely defining and documenting procedures, guidelines and protocols minimize the likelihood of operator error.

Careful and systematic implementation of all the aforementioned stages is expected to lead to desirable results for staff, current and prospective healthcare recipients (i.e., patients and the society), and the hospital. Improvement in the capabilities and satisfaction of staff and their increased commitment and accountability are some of the expected outcomes of successful implementation of the proposed accreditation model. Provision of care by motivated, responsible, and capable providers ensures the protection of patients’ rights and their satisfaction. Finally, thorough implementation of this model, coupled with effective and timely evaluations and corrective actions, will enhance the level of clinical, operational, and even financial performance of hospitals. Clinical performance appraisal refers to measuring organizational performance against clinical standards or indicators such as mortality, medication errors, blood infections and complications rates. Operational performance appraisal measures the performance of the organization against productivity indicators such as bed turnover rate in a hospital. Financial performance appraisal measures the performance of an organization in monetary terms such as profit and loss, and return on capital employed.

Weighting to model constructs was determined by national experts, with 65% devoted to enablers and 35% to results. Since in Iran, we are still at the beginning of implementing HA processes and it takes more time to achieve key performance results. The weight of each construct of HA model can be changed depending on status and importance in other countries.

### Limitations

Before interpreting our findings, several limitations should be considered. The Delphi study is a time-consuming study because it leads to the consensus of experts. The inability to access the full text of accreditation standards of some selected countries was another limitation of this study. To reduce the impact of this limitation as much as possible, the content of the hospital accreditation standards of the selected countries was obtained from related articles.

## Conclusions

A comprehensive hospital accreditation model was developed and verified. This model considers the accreditation as a system that can improve the structure, process and results. Achieving expected hospital accreditation results depends on how to manage and Accessibility and proper use of resources that named as enablers. This model can be used as a self-assessment tool to help the hospital’s managers to recognize hospital’s strengths and weaknesses. Hospital accreditation bodies can use this model to develop or revise their hospital accreditation models.

## Data Availability

The datasets used and/or analysed during the current study are available from the corresponding author on reasonable request.

## Abbreviations

HA: Hospital Accreditation
MOHME: Ministry of Health and Medical Education

## References

[1] Mosadeghrad AM (2015). Essentials of healthcare organization and management [in Persian].

[2] Mosadeghrad AM (2016). Comments on Iran hospital accreditation system. Iran J Public Health.

[3] Pomey MP, Contandriopoulos AP, François P, Bertrand D (2004). Accreditation: a tool for organizational change in hospitals?. Int J Health Care Qual Assur.

[4] Greenfield D, Braithwaite J (2008). Health sector accreditation research: a systematic review. Int J Qual Health Care.

[5] Sounan C, Lavigne G, Lavoie-Tremblay M, Harripaul A, Mitchell J, MacDonald B (2012). Using the accreditation Canada quality Worklife revalidated model to predict healthy work environments. Clin Health Promot Res Best Pract Patients Staff Commun.

[6] Shaw CD (2003). Evaluating accreditation. Int J Qual Health Care.

[7] Almasabi M, Thomas S (2017). The impact of Saudi hospital accreditation on quality of care: a mixed methods study. Int J Health Plann Manag.

[8] Oliveira JL, Magalhães AM, Bernardes A, Haddad MD, Wolff LD, Marcon SS, Matsuda LM (2019). Influence of hospital accreditation on professional satisfaction of the nursing team: mixed method study. Rev Lat Am Enfermagem.

[9] Schmaltz SP, Williams SC, Chassin MR, Loeb JM, Wachter RM (2011). Hospital performance trends on national quality measures and the association with joint commission accreditation. J Hosp Med.

[10] Mosadeghrad AM, Akbari Sari A, Yousefinezhadi T (2019). Evaluation of accreditation effects in hospitals [in Persian]. Tehran Univ Med J.

[11] El-Jardali F, Jamal D, Dimassi H, Ammar W, Tchaghchaghian V (2008). The impact of hospital accreditation on quality of care: perception of Lebanese nurses. Int J Qual Health Care.

[12] Alkhenizan A, Shaw C (2011). Impact of accreditation on the quality of healthcare services: a systematic review of the literature. Ann Saudi Med.

[13] Heuer AJ (2011). Hospital accreditation and patient satisfaction: testing the relationship. J Healthc Qual.

[14] Hosford SB (2008). Hospital progress in reducing error: the impact of external interventions. Hosp Top.

[15] Braithwaite J, Greenfield D, Westbrook J, Pawsey M, Westbrook M, Gibberd R, Naylor J, Nathan S, Robinson M, Runciman B, Jackson M (2010). Health service accreditation as a predictor of clinical and organisational performance: a blinded, random, stratified study. BMJ Qual Saf.

[16] Haj-Ali W, Karroum LB, Natafgi N, Kassak K (2014). Exploring the relationship between accreditation and patient satisfaction–the case of selected Lebanese hospitals. Int J Health Policy Manag.

[17] Ng K, Leung GK, Johnston JM, Cowling BJ (2013). Factors affecting implementation of accreditation programmes and the impact of the accreditation process on quality improvement in hospitals: a SWOT analysis. Hong Kong Med J.

[18] Sekimoto M, Imanaka Y, Kobayashi H, Okubo T, Kizu J, Kobuse H, Mihara H, Tsuji N, Yamaguchi A, Japan Council for Quality Health Care, Expert Group on Healthcare-Associated Infection Control and Prevention (2008). Impact of hospital accreditation on infection control programs in teaching hospitals in Japan. Am J Infect Control.

[19] Chen J, Rathore SS, Radford MJ, Krumholz HM (2003). JCAHO accreditation and quality of care for acute myocardial infarction. Health Aff.

[20] Andres EB, Song W, Song W, Johnston JM (2019). Can hospital accreditation enhance patient experience? Longitudinal evidence from a Hong Kong hospital patient experience survey. BMC Health Serv Res.

[21] Gimeno D, Felknor S, Burau K, Delclos G (2005). Organisational and occupational risk factors associated with work related injuries among public hospital employees in Costa Rica. Occup Environ Med.

[22] Sack C, Scherag A, Lütkes P, Günther W, Jöckel KH, Holtmann G (2011). Is there an association between hospital accreditation and patient satisfaction with hospital care? A survey of 37000 patients treated by 73 hospitals. Int J Qual Health Care.

[23] Barker KN, Flynn EA, Pepper GA, Bates DW, Mikeal RL (2002). Medication errors observed in 36 health care facilities. Arch Intern Med.

[24] Nandraj S, Khot A, Menon S, Brugha R (2001). A stakeholder approach towards hospital accreditation in India. Health Policy Plan.

[25] Mosadeghrad AM, Braithwaite J (2018). Iran hospital accreditation: Future directions. Health Care Systems: Future Predictions for Global Care, Taylor & Francis.

[26] Mosadeghrad AM, Akbari-sari A, Yousefinezhadi T (2017). Evaluation of hospital accreditation standards [in Persian]. Razi J Med Sci.

[27] Iran Health Ministry, Implementation guideline of the 4^th^ hospital accreditation survey. Ministry of Health. 2019.

[28] Mosadeghrad AM, Ghazanfari F. Iran hospital accreditation governance: Challenges and solutions payavard. 2020;14(4):311–32 [In Pesian].

[29] Ghazanfari F, Mosadeghrad AM, Jaafari Pooyan E, Mobaraki H (2021). Iran hospital accreditation standard: challenges and solution. Int J Health Plann Manag.

[30] Reisi N, Raeissi P, Sokhanvar M, Kakemam E (2019). The impact of accreditation on nurses' perceptions of quality of care in Iran and its barriers and facilitators. Int J Health Plann Manag.

[31] Moradi R, Nemati A, Bahmanziari N, Shokri A, Mohammadi M (2015). The impact of accreditation on services of Isfahan University hospitals. Health Care Manag J.

[32] Mosadeghrad AM, Akbari-sari A, Yousefinezhadi T (2017). Evaluation of hospital accreditation method [in Persian]. Tehran Univ Med J.

[33] Pourreza A, Mosadeghrad AM, Zoleikani P (2017). The impact of accreditation on the performance of hospital emergency departments. J Health Based Res.

[34] Mosadeghrad AM, Shahebrahimi SS, Ghazanfari M (2018). Exploring the relationship between accreditation and hospital performance: using data mining approach [in Persian]. J School Public Health Institute.

[35] Jaafaripooyan E, Sharifi T, Yekani Nejad MS, Esmaeili S (2018). Relationship between accreditation rank and technical efficiency of hospitals affiliated to Tehran University of Medical Sciences [in Persian]. J Hosp.

[36] Fotuhi MA, Khoshgoftar A, Bakhshande A, Karami Q, Rasti M (2018). Evaluation of viewpoint of executive management team of hospitals of Qom Province in terms of the third generation of hospital accreditation standards. Qom Univ Med Sci J.

[37] Yarmohammadian M, Shokri A, Bahmanziari N, Kordi K (2013). The blind spots on accreditation program. J Health Syst Res.

[38] Mohebbifar R, Rafiei S, Asl AM, Ranjbar M, Khodayvandi M (2017). Association between hospital accreditation and patient satisfaction: a survey in the western province of Iran. Bangladesh J Med Sci.

[39] Mosadeghrad AM, Yousefinezhadi T (2019). Evaluation of hospital accreditation implementation in Iran. Payesh.

[40] Tashayoei N, Raeissi P, Nasiripour AA (2020). Challenges of implementation of hospital accreditation in Iran: an exploratory factor analysis. J Egypt Public Health Assoc.

[41] Yousefinezhadi T, Mosadeghrad AM, Hinchcliff R, Akbari-Sari A (2020). Evaluation results of national hospital accreditation program in Iran: the view of hospital managers. J Healthcare Qual Res.

[42] Mosadeghrad AM, Nabizade Z (2018). Evaluation of Iranian hospital accreditation system [in Persian]. Payesh.

[43] Gustafson DH, Shukla RK, Delbecq A, Walster GW (1973). A comparative study of differences in subjective likelihood estimates made by individuals, interacting groups, delphi groups, and nominal groups. Organ Behav Hum Perf.

[44] Mosadegh A, Akbari-Sari A, Rahimitabar P (2020). Health system governance in Iran: a Delphi study. Sci J School Public Health Institute Public Health Res.

[45] Gale NK, Heath G, Cameron E, Rashid S, Redwood S (2013). Using the framework method for the analysis of qualitative data in multi-disciplinary health research. BMC Med Res Methodol.

[46] Glaser B, Strauss A (1976). The discovery grounded theory. Strategies for qualitative inquary. Obesvation.

[47] Powell C (2003). The Delphi technique: myths and realities. J Adv Nurs.

[48] Donabedian A (1988). The quality of care: hHow can it be assessed?. JAMA.

[49] Ghiasipour M, Mosadeghrad AM, Arab M, Jaafaripooyan E (2017). Leadership challenges in health care organizations: the case of Iranian hospitals. Med J Islam Repub Iran.

[50] Beer M (2003). Why total quality management programs do not persist: the role of management quality and implications for leading a TQM transformation. Decis Sci.

[51] Waldman DA, Lituchy T, Gopalakrishnan M, Laframboise K, Galperin B, Kaltsounakis Z (1998). A quality analysis of leadership and quality improvement. Leadersh Q.

[52] Lawrence DM, Early JF (1992). Strategic leadership for quality in healthcare. Qual Prog.

[53] Kaynak H, Hartley JL (2008). A replication and extension of quality management into the supply chain. J Oper Manag.

[54] Russel RS, Taylor BW (2006). Operations management: quality and competitiveness in a global environment, 5th ed.

[55] Hatfield M, Sonnenschein HF, Rosenberg LE (2001). Exceptional returns: the economic value of America’s investment in medical research.

[56] Murphy K, Topel R (1999). The economic value of medical research.

[57] Oakland JS (2003). Total quality management: text with cases.

[58] Akao Y (1990). Quality function deployment: integrating customer requirements into product design.

[59] Kanji GK, Asher M (1993). Total quality management process: a systematic approach.

[60] Gemme EM (1997). Retaining customers in a managed care market. Mark Health Serv.

[61] Baidoun S (2003). An empirical study of critical factors of TQM in Palestinian organizations. Logist Inf Manag.

[62] Shea CM, Howell JM (1998). Organisational antecedents to the successful implementation of total quality management: a social cognitive perspective. J Qual Manag.

[63] Deming WE (1986). Out of the crisis.

[64] Yusefi A, Kavosi Z, Heydari R, Siavashi E (2017). The barriers against strategic plan implementation from managers’ perspectives in teaching hospitals of Shiraz University of medical sciences [in Persian]. Sadra Med Sci J.

